# Immune checkpoint inhibitor-induced musculoskeletal manifestations. A multicentre prospective study

**DOI:** 10.31138/mjr.31.2.239

**Published:** 2020-04-24

**Authors:** Dimitrios Daoussis, Konstantinos Melissaropoulos, Theodoros Dimitroulas, Haralambos Andreadis, Athina Christopoulou, George Douganiotis, Thomas Makatsoris, Angelos Koutras, Panagiotis Georgiou, Haralabos Kalofonos

**Affiliations:** 1 Department of Rheumatology, Patras University Hospital, University of Patras Medical School, Patras, Greece; 2 Department of Rheumatology, Agios Andreas Hospital, Patras, Greece; 3 4th Department of Internal Medicine Hippokration Hospital, Medical School, Aristotle University of Thessaloniki, Thessaloniki, Greece; 4 3^rd^ Department of Clinical Oncology, Theageneio Cancer Hospital, Thessaloniki, Greece; 5 Oncology/Chemotherapy Unit, Agios Andreas Hospital, Patras, Greece; 6 Department of Oncology, Patras University Hospital, University of Patras Medical School, Patras, Greece

**Keywords:** Immune checkpoint inhibitors, musculoskeletal, arthritis, rheumatic, cancer immunotherapy, anti-PD-1, anti-CTLA4

## Abstract

**Background:**

Immune checkpoint inhibitors (ICI) are anti-cancer drugs that act by enhancing anti-tumour immunity. Due to their mechanism of action, they have been associated with immune related adverse events (Ir-AE), including musculoskeletal manifestations.

**Aim:**

To assess a) the prevalence, clinical and imaging (MRI) characteristics of ICI-induced musculoskeletal immune related adverse events (ir-AE) in a prospective manner, b) the potential association of musculoskeletal ir-AE with oncologic response and changes in the immune system at the level of soluble molecules (cytokines) as well as T/B cell subpopulations.

**Methods:**

This a multicentre prospective study. We plan to recruit all patients who are going to start treatment with ICI from October 2019 until October 2020 in all collaborating Oncology Departments. This study is consisted of a clinical and a laboratory arm.

**Results:**

The study is currently recruiting patients.

**Conclusions:**

We anticipate that this study will provide useful data regarding the clinical characteristics of ICI-induced musculoskeletal manifestations as well as potential predictive biomarkers.

## BACKGROUND

The use of immunotherapy in Oncology has been expanding rapidly during the last years. Immune checkpoints are molecules which act as natural brakes and prevent T cell overactivation.^1,2^ Immune checkpoint inhibitors (ICI), a novel class of effective anti-cancer agents, act by releasing these brakes leading to T cell overactivation. In this way, cytotoxic T cells become more efficient in attacking and destroying tumour cells.^3^ ICI act by stimulating the immune system and therefore they have been associated with immune related adverse events (ir-AE). Ir-AE may affect any organ and their severity ranges from mild to severe/life-threatening.^4^

Rheumatic/musculoskeletal ir-AE in the form of mild arthralgias/myalgias are relatively common and probably are not clinically significant. However, more serious musculoskeletal manifestations including inflammatory arthritis, polymyalgia rheumatica and myopathy/myositis, have been reported.^5^ The majority of studies assessing musculoskeletal ir-AE are retrospective and have reported heterogeneous results regarding prevalence and clinical characteristics. Three prospective studies have been so far published,^6,7^ including one from our department.8 There are no data on potential predictive biomarkers of musculoskeletal ir-AE.

## OBJECTIVE

This is a prospective, multicentre study aiming to assess:

- the prevalence, clinical and imaging (MRI) characteristics of ICI-induced musculoskeletal ir-AE in a prospective manner
- the potential association of musculoskeletal ir-AE with oncologic response
- changes in the immune system at the level of soluble molecules (cytokines) and T/B cell subpopulations

## STUDY DESIGN

We plan to recruit all patients who are going to start treatment with ICI from October 2019 until October 2020 in all collaborating Oncology Departments. This study is consisted of a clinical and a laboratory arm.

### Clinical arm

Screening for musculoskeletal ir-AE will be performed monthly, with the use of a specific questionnaire, by oncologists. Patients will be referred to Rheumatology Departments if they have joint swelling with no history of trauma or new onset arthralgia or myalgia with associated morning stiffness. At the initial evaluation at the Rheumatology Departments a detailed medical history will be recorded including the following: 1) age and gender, 2) type of cancer and time of diagnosis, 3) type of immunotherapy and date of treatment initiation, 4) comorbidities and drugs, 5) history of any autoimmune disease and finally 6) family history of any autoimmune disease. Patients will be subjected to a complete clinical examination and laboratory workup that includes: i) full blood count and biochemistry, ii) inflammatory markers (ESR and CRP) and iii) serology profile (RF, anti-CCP, ANA, anti-dsDNA, ENA, C3, C4, ANCA). Following the clinical assessment, patients with musculoskeletal ir-AE will be subjected to MRI of the affected area(s).

In this study, we plan to recruit 160 patients (100 from our centre and 30 from each of the 2 collaborating Departments). Based on a prevalence of 7–10%, we estimate that approximately 15 patients will develop musculoskeletal ir-AE.

We plan to assess the oncology medical record of all patients and record tumour response to ICI (progressive disease [PD]/stable disease [SD]/partial remission [PR]/complete remission CR]).^9^ A local Ethics Committee approval has been obtained from all participating centres. We plan to follow up and record data of patients with musculoskeletal ir-AE for as long as these patients have follow-up by oncologists. All participants will provide written informed consent.

### Laboratory arm

Thirty ml of heparinized venous blood will be collected from all study subjects at baseline. A part of that (5 ml) will be centrifuged and serum samples will be collected and stored in aliquots at −70° C. The rest (25 ml) will be used for peripheral blood mononuclear cell (PBMC) separation using standard methodology (Ficoll separation).

A second blood sample will be similarly collected from patients developing musculoskeletal ir-AE during their evaluation at the Rheumatology Departments. We will also obtain blood samples from 10 patients receiving ICI for 6 months and have not developed any kind of ir-AE, in a similar manner.

We plan to assess:

- the levels of cytokines related to the main T cell subpopulations Th1/Th2/Th17 (IL2, IL4, IL5, IL6, IL-10, IL12, IL17, IL21, IL23, TNF, INFγ, TGFβ). We will then compare the level of each cytokine prior to and following treatment with ICI in two patient groups:
  - patients developing musculoskeletal ir-AE,
  - and b) patients with no ir-AE (of any kind)
- specific T and B cell subpopulations using flow cytometry. We will assess Th1/Th2/Th17 defined as CD4^+^ INFγ^+^, CD4^+^ IL4+ and CD4+IL17^+^, respectively. We will also assess the following B cell subpopulations (CD19^+^CD21^low^, CD19^+^CD27^+^CD38^high^) based on recent data that changes in these cell populations may predict the development of ir-AE.^10^ We will then compare the percentage of each cell subpopulation prior to and following treatment with ICI in two patient groups a) patients developing musculoskeletal ir-AE and b) patients with no ir-AE (of any kind).

## SIGNIFICANCE OF THE STUDY

We have recently shown that musculoskeletal ir-AE mostly involve periarticular structures and that the prominent imaging finding is myo-fasciitis and not synovitis (Daoussis et al., Rheumatology, In press). Moreover, we found a strong association of musculoskeletal ir-AE with a favourable oncologic response. In this prospective, multicentre study, we aim at assessing a large number of patients treated with ICI and focus on those developing musculoskeletal ir-AE in an effort to verify our preliminary data. This will hopefully lead to a better understanding of the pathophysiology of these manifestations. Moreover, we will assess the potential association of musculoskeletal ir-AE with oncologic response. If our preliminary data are confirmed in the present study it will be important, especially for our Oncology colleagues; this will mean that patients developing these manifestations are more likely to exhibit a favourable oncologic response. Therefore, oncologists should pursue treatment with ICI despite these side effects.

The second and most important aim of this study is finding potential predictive biomarkers for ir-AE. So far there is a paucity of data on predictive biomarkers; specifically, for musculoskeletal ir-AE, there no relevant data. The discovery of predictive biomarkers for ir-AE is of importance since several ir-AE may be severe/life threatening. Oncologists need tools to identify patients at high risk for developing ir-AE; in these patients, treatment with ICI could be avoided.
